# Antioxidant properties and inhibition of angiotensin-converting enzyme by highly active peptides from wheat gluten

**DOI:** 10.1038/s41598-021-84820-7

**Published:** 2021-03-04

**Authors:** Wen-Ying Liu, Jiang-Tao Zhang, Takuya Miyakawa, Guo-Ming Li, Rui-Zeng Gu, Masaru Tanokura

**Affiliations:** 1 College of Engineering, China Agricultural University, Beijing, 100083 People’s Republic of China; 2 Beijing Engineering Research Center of Protein and Functional Peptides, China National Research Institute of Food and Fermentation Industries Co., Ltd, Beijing, 100015 People’s Republic of China; 3 College of Life Science and Technology, HuaZhong University of Science and Technology, Wuhan, People’s Republic of China; 4 Department of Applied Biological Chemistry, Graduate School of Agricultural and Life Sciences, The University of Tokyo, 1-1-1 Yayoi, Bunkyo-ku, Tokyo, 113-8657 Japan

**Keywords:** Peptides, Biomaterials - proteins

## Abstract

This study aimed to focus on the high-value utilization of raw wheat gluten by determining the potent antioxidant peptides and angiotensin I-converting enzyme (ACE) inhibitory peptides from wheat gluten oligopeptides (WOP). WOP were analyzed for in vitro antioxidant activity and inhibition of ACE, and the identification of active peptides was performed by reversed-phase high-performance liquid chromatography and mass spectrometry. Quantitative analysis was performed for highly active peptides. Five potent antioxidant peptides, Leu-Tyr, Pro-Tyr, Tyr-Gln, Ala-Pro-Ser-Tyr and Arg-Gly-Gly-Tyr (6.07 ± 0.38, 7.28 ± 0.29, 11.18 ± 1.02, 5.93 ± 0.20 and 9.04 ± 0.47 mmol 6-hydroxy-2,5,7,8-tetramethylchroman-2-carboxylic acid (Trolox) equivalent/g sample, respectively), and five potent ACE inhibitory peptides, Leu-Tyr, Leu-Val-Ser, Tyr-Gln, Ala-Pro-Ser-Tyr and Arg-Gly-Gly-Tyr (half maximal inhibitory concentration (IC_50_) values = 0.31 ± 0.02, 0.60 ± 0.03, 2.00 ± 0.13, 1.47 ± 0.08 and 1.48 ± 0.11 mmol/L, respectively), were observed. The contents of Leu-Tyr, Pro-Tyr, Tyr-Gln, Ala-Pro-Ser-Tyr, Arg-Gly-Gly-Tyr, and Leu-Val-Ser were 155.04 ± 8.36, 2.08 ± 0.12, 1.95 ± 0.06, 22.70 ± 1.35, 0.25 ± 0.01, and 53.01 ± 2.73 μg/g, respectively, in the WOP. Pro-Tyr, Tyr-Gln, Ala-Pro-Ser-Tyr, Arg-Gly-Gly-Tyr, and Leu-Val-Ser are novel antioxidative/ACE inhibitory peptides that have not been previously reported. The results suggest that WOP could potentially be applied in the food industry as a functional additive.

## Introduction

Recently, the attention of food scientists has been increasingly attracted by peptides from enzymatic hydrolysates of food proteins due to their wide range of biological activities. These hydrolysates are referred to as foodborne bioactive peptides, and the composition of amino acids and the sequences of the resulting peptide fragments are often considerably different from those of larger food proteins. Different peptide fragments from a given food protein may have different physiological activities, such as antioxidation, antihypertensive, immunomodulatory, and cholesterol-lowering effects^1–4^. Nevertheless, currently, the structures of different active peptides in oligopeptides are not well described, and there is no public library describing the structure–function relationships of peptides. Therefore, it is necessary to isolate, purify, and determine the structures of bioactive peptides with specific functions.

Many normal physiological processes cause the production of metabolites that produce reactive oxygen species (ROS), which are highly unstable because of their unpaired electrons^5^. A variety of underlying diseases in the body can be caused by these ROS, such as cardiovascular and cerebrovascular diseases, arthritis, diabetes, skin diseases, processes of aging, and even tumors^6,7^. Moreover, reductions in food quality shelf life will be caused by lipid oxidation in food products^8^. With strong antioxidant activity, the usage of artificial antioxidants is regulated strictly due to potential health risks. Therefore, continued efforts to identify safe, innovative, and economical antioxidants for use as dietary supplements and to prevent lipid oxidation in foods will be of significant benefit. Peptides with antioxidant activity can also scavenge free radicals to achieve antioxidant effects^8^. Among these peptides, the most widely known is glutathione (GSH), a tripeptide with a γ-amide bond and a thiol group consisting of glutamic acid, cysteine, and glycine. GSH has potent antioxidant activity and various other biological functions^9^. Moreover, a variety of antioxidant peptides from food protein hydrolysates, such as those of egg, ham, mushroom, and Atlantic sea cucumber, have been purified and identified^10–13^.

Hypertension is a common serious chronic disease worldwide, a risk factor of heart failure, coronary artery disease, and ischemic heart diseases. It is estimated that about one billion people in the world are infected with hypertension^14^. Angiotensin-converting enzyme (ACE), which can increase blood pressure by catalyzing the conversion of the decapeptide angiotensin I to vasoconstrictor angiotensin II, plays a vital role in regulating blood pressure in the human body^15^. As a competitive inhibitor of the active region of ACE, ACE inhibitory peptide can prevent the conversion of angiotensin I to angiotensin II, thereby partially inhibiting vasoconstriction and cholesterol secretion^16^. In addition, ACE inhibitory peptide can also prevent the degradation of vasodilator bradykinin and ensure its normal ability to cause vasodilation, thus lowering blood pressure^17^. It is urgent for patients suffering from high blood pressure to make evidence-based dietary changes. There are many reports on ACE inhibitory peptides in various food proteins, such as corn-, chicken-, fish-, and walnut-based proteins^18–21^. It has been shown that lactotripeptides (isoleucine-proline-proline [IPP] and valine-proline-proline [VPP]) have significant antihypertensive effects in clinical trials and have become recognized as blood pressure-lowering oligopeptides^22^. Many antihypertensive drugs also use the same mechanism of action, but single drugs (e.g., captopril) can afford potential risks to human health, and long-term consumption may cause serious damage^23^. Thus, finding foodborne oligopeptides with related biological functions is of importance.

Wheat gluten oligopeptides (WOP) were a mixture of small-molecular-weight peptides prepared by enzymatic digestion, separation, and purification of wheat gluten. Previous study has preliminarily demonstrated that peptides from wheat germ protein hydrolysates have a variety of physiological activities, including antioxidant, ACE-inhibitory, and anticancer activities^24^. However, the structural-activity relationship of WOP with antioxidant and antihypertensive activities remains relatively understudied. In the present study, we prepared WOP with high protein content and low molecular weights using two-step enzymatic hydrolysis and multistage separation, and isolated and identified low-molecular-weight peptides with antioxidant and antihypertensive properties. These studies may lay a theoretical foundation for the future development of natural, safe antioxidants and blood pressure-lowering functional foods.

## Results and discussion

### Chemical composition and amino acid composition of the WOP

The WOP sample contained the following components (dry basis): proteins (peptides), 94.52 ± 0.56%; lipids, 0.05 ± 0.01%; ash, 2.32 ± 0.25%; and moisture, 2.35 ± 0.23%. As expected, peptides were the main component of WOP. The dissolution of wheat gluten samples during hydrolysis and the elimination of insoluble, undigested nonprotein substances after hydrolysis led to a high protein content^25^. Nanofiltration removed most of the mineral salts, and Na^+^ and Cl^−^ caused the remaining ash.

As summarized in Table 1, WOP were rich in glutamic acid (35.23 ± 0.12%), proline (10.62 ± 0.08%), leucine (5.03 ± 0.05%), serine (4.12 ± 0.01%) and phenylalanine (3.81 ± 0.05%). Furthermore, the content ratio of essential (lysine, tryptophan, phenylalanine, methionine, threonine, isoleucine, leucine and valine) and semiessential (arginine and histidine) amino acids was 21.95% and 4.48%, respectively. A rich amino acid composition exerts a significant impact on the regulation of human physiological functions. For example, glutamate is the main excitatory neurotransmitter used at synapses in the central nervous system and exerts an important effect on maintaining nerve cell signaling^25^. As the first limiting amino acid, lysine can affect the absorption and utilization of other amino acids as well as metabolism and immune function^26^. Leucine, isoleucine, and proline are branched-chain amino acids and are important sources of metabolic energy^27^. The functional activity of a peptide is critically influenced by the type and amount of amino acids constituting its makeup. Prior work has suggested that many amino acids and their derivatives, such as glutamic acid, leucine, histidine, arginine, tyrosine, lysine, valine, alanine, cysteine, and proline, have antioxidant effects. Kong et al.^28^ reported that corn oligopeptides have a considerable antioxidant capacity, with amino acids such as leucine having strong chelation effects. In WOP, the relative content of antioxidant amino acids is as high as 65.18%, such as leucine, arginine, tyrosine, cysteine, valine, alanine and other amino acids, potentially leading to chelation of metal ions, so WOP may be a natural substance with antioxidant activity. In addition, according to previous studies, many ACE inhibitory peptides contain valine, alanine, leucine, tyrosine, and phenylalanine^29^, which suggests that WOP may contain ACE inhibiting peptides and have potential ACE inhibiting activity.

**Table 1:** Amino acid composition (dry basis) of WOP (%)^a^.

Amino acids	Content^b^	Amino acids	Content^b^
Alanine	2.15 ± 0.02	Lysine	1.36 ± 0.06
Arginine	2.79 ± 0.06	Methionine	1.16 ± 0.04
Aspartic acid^c^	2.81 ± 0.03	Phenylalanine	3.81 ± 0.05
Cysteine	0.84 ± 0.04	Proline	10.62 ± 0.08
Glutamic acid^d^	35.23 ± 0.12	Serine	4.12 ± 0.01
Glycine	3.00 ± 0.03	Threonine	2.15 ± 0.02
Histidine	1.69 ± 0.05	Tryptophan	0.60 ± 0.05
Isoleucine	2.60 ± 0.03	Tyrosine	2.38 ± 0.05
Leucine	5.03 ± 0.05	Valine	3.09 ± 0.02

^a^Data correspond to the average and standard deviation of three independent experiments.

^b^The content of amino acids in WOP.

^c^Aspartic acid + asparagine.

^d^Glutamine acid + glutamine.

### Relative molecular weight distribution of WOP

**Figure 1:**
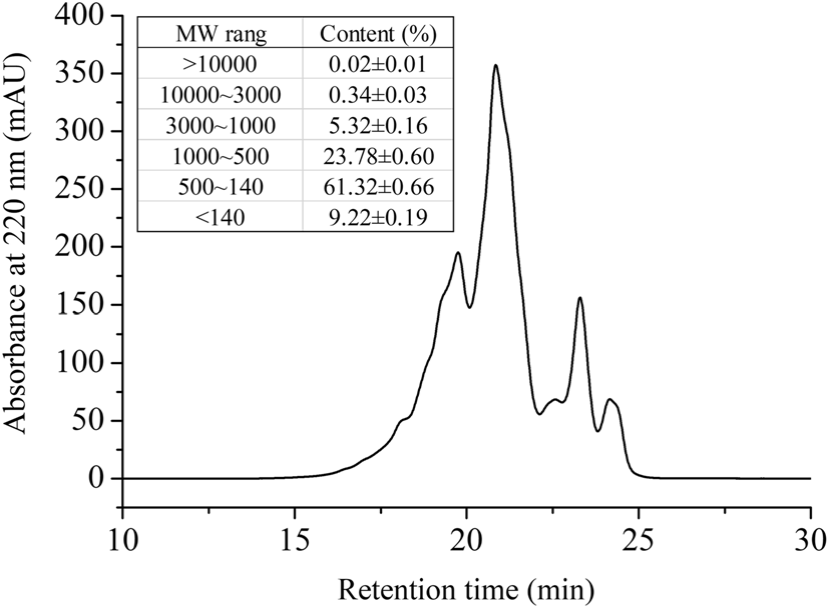
Chromatogram of WOP showing the molecular weight (MW) distribution. The inset shows the contents of peptides with different classes of MW ranges in WOP.

Size exclusion chromatography with a high-performance liquid chromatography (HPLC) system was applied to analyze the molecular weight distribution of WOP. According to the results (Fig. 1), peptides with molecular weights below 1000 (94.32%) dominated WOP. Most of the peptides in WOP (61.23%) were in the molecular weight range of 500–140 and were mainly di- and tripeptides. According to reports, di- and tripeptides are actively transported in intestinal epithelial cells by specific peptide transporters (e.g., PepT1, PepT2), and amino acid residues of di- and tripeptides are systemically absorbed faster than free amino acids^30,31^.

These findings indicate that a two-step enzymatic hydrolysis followed by a multistep separation may efficiently remove large peptides or undigested proteins and produce oligopeptides. This method of enzymatic hydrolysis has also been carried out in past studies with good success^32,33^. The molecular structure and weight highly exerted an influence on the functional characteristics of peptides, which are greatly affected by processing conditions. It is widely known that most ACE inhibitory peptides from food proteins have relatively low molecular weights, most of which are short peptides less than 1000 Da^34^. Moreover, previous studies have demonstrated that compared with relatively large peptides, small peptides have higher antioxidant activities, and several di- and tripeptides have shown greater antioxidant activities than those their constituent amino acids^35,36^. According to recent advances in oxidant-antioxidant test systems, the effects of small peptides were greater than those of larger peptides and proteins^37^.

### Antioxidant properties of WOP

As an enzymatic hydrolysate, WOP showed strong scavenging ability against 1,1-diphenyl-2-picrylhydrazyl (DPPH), hydroxyl and 2,2′-azinobis (3-ethylbenzothiazoline-6-sulfonic acid) diammonium salt (ABTS) free radicals. The concentration for 50% of maximal effect (EC_50_) of DPPH and hydroxyl free radical scavenging activity were 1.52 ± 0.09 and 6.23 ± 0.18 mg/mL, respectively, and ABTS free radical scavenging activity was 1.23 ± 0.03 mmol Trolox equivalent (TE)/g sample. Zhang et al. hydrolyzed wheat germ protein by Alcalase 2.4 L to obtain wheat germ peptides (molecular weight < 1000 Da), and the EC_50_ value for DPPH were found to be 2.77 mg/mL^38^. Karami et al. evaluated the DPPH free radical scavenging activity of three hydrolysates of wheat germ protein prepared with Alcalase, pepsin or proteinase K, and the EC_50_ values were 1.56, 1.78 and 1.83 mg/mL, respectively^24^. Compared with the above studies, the DPPH free radical scavenging activity of WOP in this study was slightly higher. Additionally, The EC_50_ value of WOP for hydroxyl free radicals was better than those of peptides obtained from defatted wheat germ (6.04 mg/mL)^39^. Besides, WOP exhibited a high oxygen radical absorption capacity (ORAC) value (1216.87 ± 11.51 mmol TE/g sample) (Table 2). The high antioxidant peptides obtained by hydrolyzing chicken egg whites with different proteases had an ORAC value between 900–1300 mmol Trolox equivalent (TE)/g sample, which is close to WOP^40^. Moreover, these antioxidant properties of WOP showed a strong dose-dependent relationship. The results revealed that two-step enzymatic hydrolysis was an effective approach to obtain an antioxidant hydrolysate from wheat gluten. Given its apparent antioxidant properties, amino acids or peptides contained in WOP act as electron donors and react with free radicals to generate stable products and end radical chain reactions. Among the four types of antioxidant assay system results, the ABTS free radical scavenging activity of WOP was remarkably strong. Moreover, the ABTS experiment was seemingly very effective in assessing antioxidant capacity. Therefore, the ABTS free radical antioxidant assay was used in further experiments.

**Table 2:** Antioxidant activities of WOP using four types of assay systems.

Sample	Radical scavenging activity			
	DPPH^a^	Hydroxyl^a^	ABTS^b^	ORAC^c^
WOP	1.52 ± 0.09	6.23 ± 0.18	1.23 ± 0.03	1216.87 ± 11.51

^a^EC_50_ value (mg/mL), defined as the concentration of samples needed to scavenge 50% of the free radicals.

^b^mmol Trolox equivalent (TE)/g sample.

^c^μmol TE/g sample.

### ACE inhibitory activity of WOP

**Figure 2:**
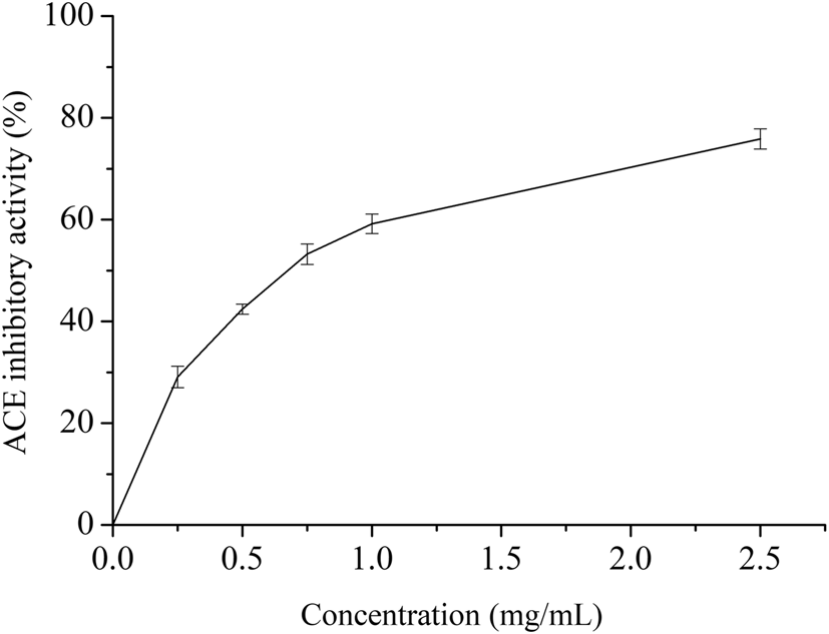
Angiotensin I-converting enzyme (ACE) inhibitory activity of WOP at different concentrations. The data are the average of triplicate experiments. Error bars show the standard deviations.

As demonstrated in Fig. 2, the ACE inhibition of WOP gradually increased with increasing concentration. The inhibition rate was 77% at a concentration of 2.5 mg/mL, and the half maximal inhibitory concentration (IC_50_) values was 0.68 ± 0.03 mg/mL. According to Bougatef et al.^41^, sardine protein hydrolysate had an IC_50_ value of 1.2 mg/mL for ACE. Gu et al.^33^ showed that the IC_50_ value of oligopeptides derived from black-bone silky fowl for ACE was 2.86 mg/mL. Moreover, after measuring the ACE inhibitory activity of 48 marine protein hydrolysates, He et al.^42^ found IC_50_ values ranging from 0.17 to 501.7 mg/mL. Compared with these data, WOP had a relatively high ACE inhibitory activity. As there were many kinds of peptides in WOP, we speculated that some of the peptides in WOP may be competitive inhibitors with greater affinity for the ACE active region than WOP. According to studies^32,34^, the ACE inhibitory activity of peptides may be connected with the length of their peptide chains. The molecular weights of ACE inhibitory peptides are relatively low, generally less than 1000. Furthermore, hydrophobic amino acids in the C-terminus of antihypertensive peptides are significant for the activity of antihypertensive peptides^33^. WOP may contain a relatively large amount of peptides that can inhibit ACE. Therefore, there is strong reason to continue to identify and purify ACE inhibitory peptides from WOP.

### ABTS free radical scavenging and ACE inhibitory characteristics of reversed-phase high performance liquid chromatography (RP-HPLC) fractions

**Figure 3:**
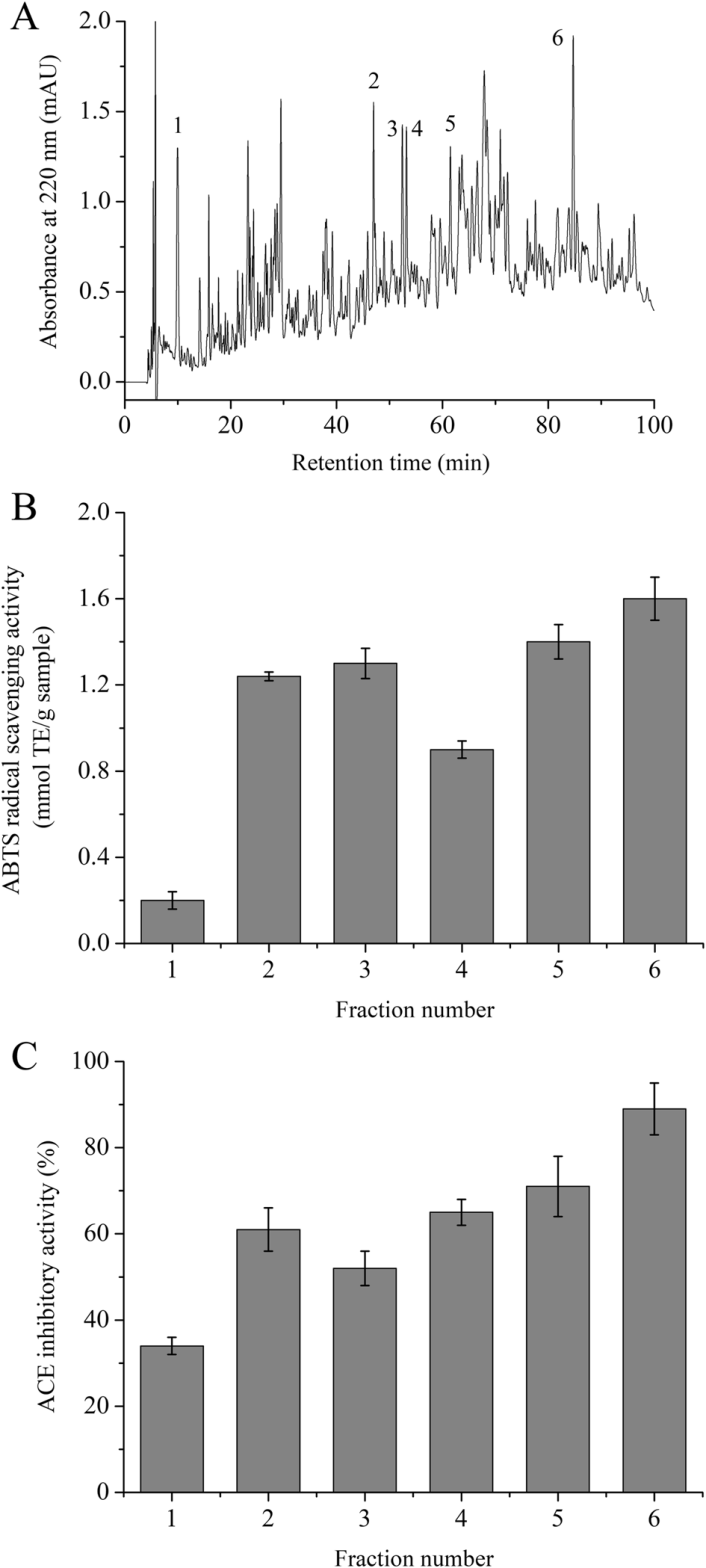
(**A**) Elution profile of WOP separated by RP-HPLC on an XBridge BEH130 C18 column (4.6 mm × 250 mm) at a flow rate of 0.6 mL/min with a linear gradient of solvent A (0.1% TFA in Milli-Q water, v/v) and solvent B (80% acetonitrile with 0.1% TFA, v/v). Six fractions were collected and designated fractions 1–6. (**B**) ABTS free radical scavenging activity of the collected fractions from the RP-HPLC system. The results are expressed as mmol Trolox equivalent (TE)/g sample. (**C**) ACE inhibitory activity of the collected fractions from the RP-HPLC system at a concentration of 0.7 mg/mL. Error bars indicate the standard deviation (n = 3).

An RP-HPLC column was applied to fractionate WOP to determine the possible impacts of peptide composition on antioxidant activity and ACE inhibitory activity. Figure 3A shows the elution profiles of WOP. According to peak shape, height, area, and resolution, six component peaks were collected, fractions 1–6, where each component peak did not necessarily represent a single peptide. Repeated chromatography was used to separately collect six fractions, and their ABTS free radical scavenging and ACE inhibitory activities were determined. According to Fig. 3B, fractions 2–6 expressed equal or even higher ABTS radical scavenging activity (0.91–1.62 mmol TE/g sample) than that of WOP (1.23 ± 0.03 mmol TE/g sample), but fraction 1 had a lower activity.

The ACE inhibition results were similar to those above, where fractions 2–6 exhibited higher ACE inhibitory activities (61.21–89.36%) than that of WOP, and fractions 1 showed a lower ACE inhibitory activity (34.18 ± 2.06%) at the concentration of 0.7 mg/mL (Fig. 3C). The inhibitory activities of fractions 5 and 6 were 1.39 and 1.74 times that of WOP, respectively.

According to these results, some peptides in certain fractions may have relatively higher ABTS free radical scavenging activity and ACE inhibition (or are present at a higher concentration) than those of peptides in other fractions. Therefore, for further identification and screening of active peptides, fractions 1–6 were all chosen.

### Purification and recognition of antioxidative peptides and ACE inhibitory peptides in WOP

For fractions 1–6, an electrospray ionization-quadrupole time-of-flight mass spectrometer (ESI-Q-TOF2) was used to recognize active peptides, resulting in the identification of 40 active peptides from WOP whose amino acid sequences and relative molecular masses are listed in Table 3. These peptides were composed of 2–6 amino acids, which was consistent with the relative molecular mass analysis described above. It was reported that antioxidant and ACE inhibitory activities of peptides were highly influenced by peptide molecular weight. Peng et al.^43^ reported that the 100–2800 Da fraction of peptides purified from the hydrolysate of whey protein had the strongest antioxidant activity. Saiga et al.^44^ studied the peptides in hydrolyzed chicken breast muscle extract, and the result indicated that the small peptides (< 1000 Da) showed higher activity than those with molecular weight of > 1000 Da. The peptides identified in our study were all small peptides with molecular weight of < 1000 Da. Therefore, it was expected that the peptides identified in this study would exhibit antioxidant and ACE inhibitory activities. Selections of the peptides for ABTS free radical scavenging activity and ACE inhibitory activity assays were based on previous experience and related references. Therefore, we conducted a preliminary analysis of the structural characteristics and amino acid composition of antioxidant peptides and ACE inhibitory peptides. Specifically, according to previous studies, some di- and tripeptides with aromatic amino acid residues (Tyr or Trp) and peptides containing Arg, Pro, Gln, or Asn are highly likely to have strong antioxidant activity^1,10,12^. In addition, peptides carrying a Tyr, Phe, Trp, Pro, or a hydrophobic amino acid residue at the C-terminus have strong ACE inhibitory activity^45^. On the basis of these reports, we selected and synthesized 15 peptides as potential candidates (Table 4) with these preferred characteristics from fractions 2–6 for further testing to determine their ABTS free radical scavenging activity and ACE inhibitory activity.

**Table 3:** Identification of peptides by quadrupole time-of-flight mass spectrometry (Q-TOF2) of WOP fractions collected from reversed-phase high-performance liquid chromatography (RP-HPLC).

RP-HPLC fraction^a^	Amino acid sequence^b^	Observe mass	Calculated mass^c^
1	Gln-Val-Ser-Gln (QVSQ)	461.22	460.49
1	Thr-Phe-Asn (TFN)	381.14	380.40
1	Thr-Thr-Gly-His (TTGH)	415.20	414.42
1	Gly-Pro-Gly-Val (GPGV)	329.00	328.37
1	His-Gly-Ala-Gln (HGAQ)	412.19	411.42
1	Gln-Arg-Gln (QRQ)	431.22	430.46
1	Val-Asn (VN)	232.12	231.25
2	Thr-Phe-Gly-Gln (TFGQ)	452.18	451.48
2	Gly-Thr-Cys-Gly-Ala-Gly (GTCGAG)	465.17	464.50
2	Ala-Pro-Ser-Tyr (APSY)	437.20	436.47
2	Thr-Thr-Gly-Ala (TTGA)	348.99	348.36
2	Thr-Phe-Gln (TFQ)	395.16	394.43
2	Thr-Pro-His-Gln (TPHQ)	482.19	481.51
2	Ala-Ala-Pro-Gln (AAPQ)	386.19	385.42
3	Gly-Gly-Asp-Asn-Pro (GGDNP)	459.69	458.43
3	Lys-Asn-Phe (KNF)	408.21	407.47
3	Met-Leu-Gln (MLQ)	391.19	390.50
3	Ile-Leu-Arg (ILR)	401.29	400.52
3	Pro-Tyr (PY)	279.13	278.31
4	Lys-Pro-His-Pro (KPHP)	478.28	477.56
4	Pro-Leu-Arg (PLR)	385.27	384.48
4	Asp-Val-Thr-Gln (DVTQ)	462.22	461.47
4	Pro-Glu-Thr-Gln (PETQ)	474.21	473.48
4	Arg-Gly-Gly-Tyr (RGGY)	452.23	451.48
4	Pro-Gln (PQ)	244.14	243.26
4	Leu-Tyr (LY)	295.17	294.35
4	Pro-Gln-Ala-Gly-Gln (PQAGQ)	500.26	499.52
5	Gln-Pro-His-Pro (QPHP)	478.28	477.52
5	Asp-Ala-Asp-Thr (DADT)	421.16	420.38
5	Phe-Pro-Gln (FPQ)	391.22	390.44
5	Ser-Gly-Asn-Pro-Gln (SGNPQ)	502.24	501.50
5	Glu-Pro-Arg (EPR)	401.17	400.44
5	Gly-Gly-Asn-Gln (GGNQ)	375.18	374.35
6	Arg-Thr-His (RTH)	413.28	412.45
6	Ile-Leu-Pro-Arg (ILPR)	498.37	497.64
6	Pro-Phe-Ala-Leu (PFAL)	447.23	446.55
6	Glu-Ala-His (EAH)	356.19	355.35
6	Phe-Arg (FR)	322.13	321.38
6	Tyr-Gln (YQ)	310.23	309.32
6	Leu-Val-Ser (LVS)	318.14	317.39

^a^Fractions are indicated in Fig. 3

^b^Three-letter representations of amino acids, and single-letter representations in parentheses.

^c^Average mass.

**Table 4:** Synthesis of 15 preferred peptides.

RP-HPLC fraction^a^	Amino acid sequence^b^
1	Gln-Arg-Gln (QRQ)
1	Val-Asn (VN)
2	Ala-Pro-Ser-Tyr (APSY)
3	Lys-Asn-Phe (KNF)
3	Ile-Leu-Arg (ILR)
3	Pro-Tyr (PY)
4	Arg-Gly-Gly-Tyr (RGGY)
4	Pro-Gln (PQ)
4	Leu-Tyr (LY)
5	Gln-Pro-His-Pro (QPHP)
5	Phe-Pro-Gln (FPQ)
6	Ile-Leu-Pro-Arg (ILPR)
6	Phe-Arg (FR)
6	Tyr-Gln (YQ)
6	Leu-Val-Ser (LVS)

^a^Fractions are indicated in Fig. 3

^b^Three-letter representations of amino acids and single-letter representations in parentheses.

**Figure 4:**
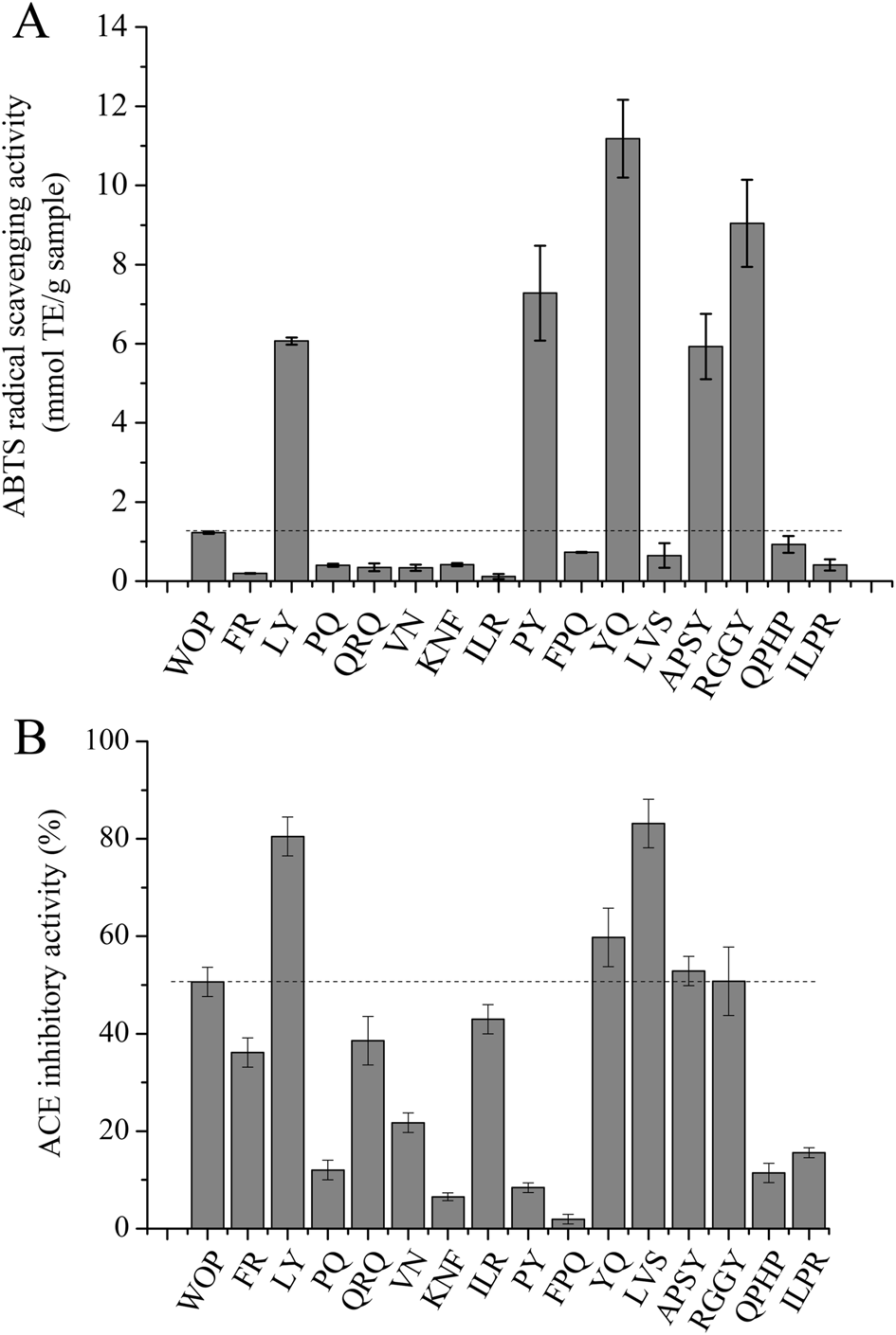
ABTS free radical scavenging activity (**A**) and angiotensin I-converting enzyme (ACE) inhibitory activity (**B**) of 15 peptides identified from fractions 2–6 by quadrupole time-of-flight (Q-TOF2) mass spectrometry. The concentration of the peptides used for ACE inhibitory activity analysis was 0.7 mg/mL. Error bars indicate the standard deviation (n = 3). TE stands for Trolox equivalent.

Total antioxidant capacities can be assessed conveniently by virtue of the ABTS experiment. Figure 4A shows the ABTS free radical scavenging capacity, in which five peptides (Leu-Tyr, Pro-Tyr, Tyr-Gln, Ala-Pro-Ser-Tyr, Arg-Gly-Gly-Tyr) had extremely strong performance. Their ABTS free radical scavenging activities were 6.07 ± 0.38, 7.28 ± 0.29, 11.18 ± 1.02, 5.93 ± 0.20 and 9.04 ± 0.47 mmol Trolox equivalent (TE)/g sample, respectively. The free radical scavenging activity was 4.93, 5.92, 9.08, 4.82, and 7.35 times the WOP activity for these peptides, respectively, and the other peptides had weaker ABTS free radical scavenging abilities. Although some peptides do not exhibit strong ABTS free radical scavenging activity, we are unsure if they have synergistic effects with other peptides to enhance the overall antioxidant activity of WOP. On the other hand, based on these results, we were confident that at least five peptides (Leu-Tyr, Pro-Tyr, Tyr-Gln, Ala-Pro-Ser-Tyr and Arg-Gly-Gly-Tyr) in WOP are major contributors to its ABTS free radical scavenging activities. The active sites and groups they exposed may be able to act as electron donors, stop the chain reaction of free radicals, and ultimately achieve antioxidant effects^33^. Beermann et al.^46^ isolated and purified soy peptides and found that the dipeptide Leu-Tyr has excellent antioxidant effects, which was in good agreement with our experiments herein. To the best of our knowledge, the other four peptides (Pro-Tyr, Tyr-Gln, Ala-Pro-Ser-Tyr, Arg-Gly-Gly-Tyr) have not been previously reported as antioxidant active peptides. Chen et al.^47^ previously speculated that short peptides with aromatic amino acid residues (Trp or Tyr) may have potent antioxidant activity. The C-termini of four of the five antioxidant peptides identified in the present study were Tyr. Saito et al.^48^ reported that the tripeptide Tyr-His-Tyr, separated from soy protein hydrolysate, has strong antioxidant capacity and great synergistic effects with phenolic antioxidants (e.g., BHA and δ-tocopherol). Interpreted by the special abilities of phenolic and indolic groups, the antioxidant activities of Trp and Tyr may act as hydrogen donors. With longer lifetimes than those of simple peroxy radicals, much more stable phenoxyl and indoyl radicals inhibit any reverse reaction or the propagation of the radical-mediated peroxidizing chain reaction^48^. In addition, oxidation of specific sites may be due to the binding of metal ions or other starting materials at specific sites on the polypeptides. The selective modification of a peptide to His, Pro, Met, Cys, Arg, Lys, and Trp residues may also be related to its antioxidant activity^48^.

Figure 4B shows that 15 peptides exhibited different ACE inhibitory activities at 0.7 mg/mL (ranging from 1.94 to 83.13%). Among them, five peptides, Leu-Tyr, Leu-Val-Ser, Tyr-Gln, Ala-Pro-Ser-Tyr, and Arg-Gly-Gly-Tyr, attracted our attention due to their increased inhibitory activity against ACE compared to that of WOP. At this concentration, their activities were 1.59, 1.64, 1.18, 1.04, and 1.01 times that of WOP, respectively. Furthermore, we determined the IC_50_ values of the five active peptides: Leu-Tyr (0.31 ± 0.02 mmol/L), Leu-Val-Ser (0.60 ± 0.03 mmol/L), Tyr-Gln (2.00 ± 0.13 mmol/L), Ala-Pro-Ser-Tyr (1.47 ± 0.08 mmol/L) and Arg-Gly-Gly-Tyr (1.48 ± 0.11 mmol/L). The IC_50_ values of the five active peptides were calculated to be 0.09, 0.19, 0.62, 0.64 and 0.67 mg/mL, respectively, in the units of mg/mL, which were 7.56, 3.58, 1.10, 1.06, and 1.01 times that of WOP (0.68 ± 0.03 mg/mL), respectively. To the best of our knowledge, there is no report that the three peptides Leu-Val-Ser, Ala-Pro-Ser-Tyr and Arg-Gly-Gly-Tyr have ACE inhibitory activity. However, there are reports that the dipeptide Leu-Tyr had ACE inhibitory activity (IC_50_ = 38.5 μM) and could lower blood pressure^49,50^. In addition, Leu-Tyr and Leu-Val-Arg, previously isolated from sardine muscle hydrolysate, have good ACE inhibitory effects^41^. The dipeptide Tyr-Gln was also already known to be isolated from buckwheat as an ACE inhibitory peptide (IC_50_ = 628 μM)^51^. In the study of Balti et al.^52^, Ala-His-Ser-Tyr isolated from cuttlefish was found to have ACE inhibitory activity (IC_50_ = 11.6 μM). Interestingly, the structure of Ala-Pro-Ser-Tyr found in the present study was similar to that of Ala-His-Ser-Tyr. Therefore, it was inferred that these two peptide sequences may result in good binding to ACE. For Arg-Gly-Gly-Tyr, another similar tetrapeptide, Tyr-Gly-Gly-Tyr, was found to be an effective ACE inhibitor in vitro by Saito et al.^53^. In addition, the digested product of Tyr-Gly-Gly-Tyr, Gly-Gly-Tyr , a tripeptide, also exerted good inhibitory activity (IC_50_ = 1.3 μM), as shown by Shamloo et al.^54^. To a lesser extent, the released amino acid tyrosine can also exhibit ACE inhibitory activity on its own^55^. In the present study, Arg-Gly-Gly-Tyr, which has the Gly-Gly-Tyr sequence, displayed potent ACE inhibitory activity. Its activity may be due to the preferred amino acid sequence (Gly-Gly-Tyr) or amino acid (tyrosine) and their synergistic effects with the amino acid arginine at the N-terminus. Regarding the association between the structure and activity of ACE inhibitory peptides, Ryan et al.^56^ suggested that hydrophobic, branched-chain or aromatic amino acids were important components of ACE inhibitory peptides, while hydrophilic amino acids had weak ACE inhibitory activity, as they were incompatible with the ACE active site. Cheung et al.^57^ noted that active peptides tended to have Pro, Phe, or Tyr at the C-terminus and Val or Ile at the N-terminus. Some ACE inhibitory peptides with similar structures at the C/N termini, such as the lactotripeptides Val-Pro-Pro and Ile-Pro-Pro in yogurt, Ile-Ala-Pro in gliadin hydrolysate, Ala-Pro and Val-Arg in salmon hydrolysate, Tyr-Ala-Pro and Val-Ile-Ile in cuttlefish hydrolysate, and Ile-Gln-Pro in algae, have been isolated and identified^22,58–61^. Among these peptides, it has been shown that lactotripeptides Val-Pro-Pro and Ile-Pro-Pro have good antihypertensive effects in clinical trials. However, the nature of the C/N terminal amino acids and whether they contain hydrophobic amino acids may not connect with the ACE inhibitory activity of short peptides. Despite the small number of amino acids in oligopeptides, simple secondary structures are formed due to the interactions between them and affect competition for the ACE active region. The antihypertensive drug captopril is synthesized according to the proline configuration^62^. The ACE inhibitory peptide is a competitive inhibitor with strong affinity to the active region of ACE. Its affinity with ACE is stronger than that of angiotensin I or bradykinin, and it is not easy to release from the ACE binding region, thus hindering the two biochemical reaction processes of catalytic hydrolysis of angiotensin I into angiotensin II and catalytic hydrolysis of bradykinin into inactivated fragment, thus lowering blood pressure^16,17^.

### Quantitative analysis of active peptides

Five antioxidant peptides and five ACE inhibitory peptides were identified from WOP. In particular, four peptides, Leu-Tyr, Tyr-Gln, Ala-Pro-Ser-Tyr and Arg-Gly-Gly-Tyr, had antioxidant and ACE inhibitory activities. High-performance liquid chromatography-tandem mass spectrometry (HPLC–MS/MS) was applied to estimate the concentration of highly active peptides in WOP. The results showed that the contents of Leu-Tyr, Pro-Tyr, Tyr-Gln, Ala-Pro-Ser-Tyr, Arg-Gly-Gly-Tyr, and Leu-Val-Ser were 155.04 ± 8.36, 2.08 ± 0.12, 1.95 ± 0.06, 22.70 ± 1.35, 0.25 ± 0.01, and 53.01 ± 2.73 μg/g of WOP, respectively (Table 5). Interestingly, it was found that four peptides have both good antioxidant activity and ACE inhibitory activity. These peptides can potentially be incorporated into functional foods for antioxidation or to partially address high blood pressure. In this study, our findings were limited in vitro. It was not clear whether the identified active peptides function in vivo. Therefore, further studies are needed to evaluate the in vivo effects of these active peptides. Previous studies have reported that the bioactivity of low-molecular-weight peptides can be retained during gastrointestinal digestion^32,33^. The bioavailability and metabolism of active peptides will also be further studied to better understand the multifunctionality of peptides.

**Table 5:** Quantitative analysis of active peptides in WOP.

Peptide sequence	Content (μg/g)
Leu-Tyr (LY)	155.04 ± 8.36
Pro-Tyr (PY)	2.08 ± 0.12
Tyr-Gln (YQ)	1.95 ± 0.06
Ala-Pro-Ser-Tyr (APSY)	22.70 ± 1.35
Arg-Gly-Gly-Tyr (RGGY)	0.25 ± 0.01
Leu-Val-Ser (LVS)	53.01 ± 2.73

## Conclusion

WOP has good antioxidant activity and ACE inhibitory activity. We identified six highly active peptides (Leu-Tyr, Pro-Tyr, Tyr-Gln, Ala-Pro-Ser-Tyr, Arg-Gly-Gly-Tyr, and Leu-Val-Ser) from WOP, four of which have dual activities, most of which were novel active peptides. This study expanded the potential types of peptides exerting antioxidant and antihypertensive activities. WOP and these active peptides may be beneficial for the future development of physiologically functional foods. The in vivo effects of each peptide and the mechanism by which these active peptides carry out their effect will be researched by further studies.

## Materials and methods

### Materials and reagents

Wheat gluten was obtained from CF Haishi Biotechnology Ltd. Co. (Beijing, China) and kept in sealed plastic bags at room temperature until use. Alcalase 2.4L (2.4 AU/g) was purchased from Novo Nordisk (Copenhagen, Denmark). Protex 7L (1600 AU/g) and α-amylase (26,000 RAU/g) were bought from Genencor Division of Danisco (Rochester, NY, USA). Fluorescein sodium salt, Trolox, DPPH, 2,2′-azobis(2-methylpropionamidine) dihydrochloride (AAPH), hippuryl-histidyl-leucine (HHL), hippuric acid (HA), and ACE (from rabbit lung) were obtained from Sigma-Aldrich Co. (St. Louis, MO, USA). The ABTS assay kit was supplied by Beyotime Biotechnology (Haimen, China). Acetonitrile and methanol were provided by Fisher Scientific (Pittsburgh, PA, USA). Trifluoroacetic acid (TFA) was supplied by Alfa Aesar (Ward Hill, MA, USA). Synthesized peptides were obtained from Scilight Biotechnology Co., Ltd (Beijing, China). The purity of the synthesized peptides was more than 98% according to HPLC analysis. All other chemicals were of analytical grade.

### Enzymolysis and preparation of WOP

Wheat gluten was suspended with distilled water at a ratio of 1:10 (w/w) by stirring in a homogenizer (Donghua Homogenizer Factory, Shanghai, China) at 25,000 × g for 10 min. The slurry was digested with α-amylase using an enzyme-to-substrate protein ratio of 1:100 (w/w) at pH 5.0 and a temperature of 75 °C for 1 h to hydrolyze the starch in wheat gluten. The hydrolysate was then centrifuged at 3000 × g (LG10-2.4A, Beijing LAB Centrifuge Co., Ltd., China) for 10 min to remove nonprotein substances such as carbohydrates. The insoluble protein precipitate was recovered and resuspended with distilled water in the homogenizer with the same procedures as above. Two-step enzymolysis was adopted for the resulting slurry of highly pure proteins. The first hydrolysis was performed with Alcalase 2.4 L (enzyme/substrate ratio of 1:100 [w/w]) at pH 8.5 at 55 °C for 2 h, and then the second hydrolysis was performed with Protex 7L for another 2 h at pH 6.5 at 60 °C at an enzyme/substrate ratio of 1:100 (w/w). The pH value was kept unchanged during the process of hydrolysis by adding 1.0 mol/L HCl or NaOH. Afterwards, hydrolysis was ceased by heating at 100 °C for 10 min. The hydrolysate was centrifuged at 3,000 × g using the same centrifuge as above for 15 min. Molecular weight cut-off (MWCO) ceramic membranes of 10,000 and 1000 Da were applied to filter the supernatant successively to obtain a filtrate containing oligopeptides. Next, the filtrate with oligopeptides was subjected to nanofiltration to remove mineral salts and free amino acids. An R-151 rotary evaporator (BUCHI Co., Ltd., Switzerland) was used to concentrate the resulting filtrate, and activated carbon was applied to decolor it. Then, an L-217 Lab spray dryer (Beijing Laiheng Lab-Equipments Co., Ltd., Beijing, China) was employed to spray dry the concentrated oligopeptide solution to obtain oligopeptide powder. Spray drying was carried out at 25 mL/min feed temperature, 14 kPa feed rate, 135 °C inlet air temperature and 20 °C inlet air pressure.

### Determination of chemical composition and amino acid composition

The methods of the Association of Official Analytical Chemists were employed to explore the WOP chemical composition. Protein, lipids, ash, moisture, and contents of WOP were determined according to AOAC Official Method 979.09, 996.06, 923.03, and 2001.12^63^. An 835-50 automatic amino acid analyzer (Hitachi, Ltd., Tokyo, Japan) was applied to determine the amino acid composition in accordance with the approaches of Yang et al.^64^.

### Determination of molecular weight distribution

An LC-20A high-performance liquid chromatography (HPLC) system (Shumadzu, Kyoto, Japan) configured with a TSK gel G2000 SWXL column (300 mm × 7.8 mm, Tosoh, Tokyo, Japan) was used to assess molecular weight distribution. Acetonitrile/water (45:55, v/v) with 0.1% (v/v) trifluoroacetic acid acted as the mobile phase. The elution of samples was performed at a flow rate of 0.5 mL/min and monitored at 220 nm and 30 °C. Molecular weight standards were subjected to tripeptide GGG (MW 189), tetrapeptide GGYR (MW 451), bacitracin (MW 1450), aprotinin (MW 6500), and cytochrome C (MW 12,500) (Sigma Chemical Co., St. Louis, USA).

### DPPH free radical scavenging activity assay

The approach of Kedare et al.^65^ was applied to measure the DPPH free radical scavenging ability of the samples. Briefly, 100 μL of 0.1 mmol/L DPPH in 95% ethanol was added to 100 μL of each sample at various solid concentrations. After shaking, the mixture stood for 30 min in the dark at room temperature, and then a Spectra MR multimode reader (DYNEX Technologies, Inc., Chantilly, VA, USA) was applied to assess the absorbance at 517 nm (A_S_). Samples in the control experiment (A_C_) were replaced by ethanol, and a blank excluding DPPH (A_B_) was prepared as above. The calculation of scavenging activity was subject to [1 − (A_S _− A_B_)/(A_C _− A_B_)] × 100%.

### Hydroxyl free radical scavenging activity assay

The modified approach of Smirnoff and Cumbes^66^ was applied to measure the hydroxyl radical scavenging effect. The reaction system, which contained 200 μL of 2.3 mmol/L FeSO_4_, 200 μL of 2.3 mmol/L sodium salicylate in ethanol, 100 μL of samples at different concentrations, and 100 μL of 2.2 mmol/L H_2_O_2_, was incubated for 1 h at 37 °C in a water bath. The absorbance of 200 μL of the resulting solution was measured at 510 nm (A_S_) after incubation. Samples in the control experiments (A_C_) were replaced by distilled water. The scavenging activity of the samples was calculated as [(A_C _− A_S_)/A_C_] × 100%.

### ABTS free radical scavenging activity assay

The ABTS assay kit instructions were applied to assess the ABTS free radical scavenging activity. Briefly, the 7 mmol/L ABTS stock solution in 2.45 mmol/L potassium persulfate solution constituted the stock solution of ABTS free radicals, and the resulting mixture was placed in the dark at room temperature for 16 h. The ABTS free radical solution was diluted with distilled water to an absorbance of 0.70 ± 0.05 at 734 nm. The diluted ABTS free radical solution (200 μL) was mixed with the sample (10 μL), and the absorbance was recorded at 734 nm after 10 min (distilled water was used as the control). The final scavenging activity was expressed as mmol TE/g sample^33^.

### ORAC-based assay

For the ORAC-based assay, the approach of Schauss et al.^67^ with minor modifications was applied. First, the sample was diluted to the appropriate concentration with 75 mmol/L phosphate buffer solution (PBS) at pH 7.4, and then in a black 96-well plate, 100 μL of fluorescein (fluorescent indicator, 0.8 μmol/L) was added to 25 μL of sample. For the preparation of the standard curve, 25 μL of Trolox standard (6.25, 12.5, 25, 50, 75, 10, 250, 500 μmol/L) was used as a positive control. After a 20-min incubation at 37 °C, 75 μL of 150 mmol/L AAPH was added. The excitation and emission wavelengths of the fluorescence microplate reader (SpectraMax i3x multimode reader, Molecular Devices, LLC., San Jose, CA, USA) were 485 and 530 nm, respectively, and the fluorescence was measured every 2 min. The total read time was 150 min, and PBS was used as a blank control. In addition, a control without AAPH was used.

We calculated the relative fluorescence value and the integral area (AUC) under the fluorescence decay curve with and without antioxidant. Then, we subtracted the control AUC without the antioxidant to calculate the protective area of the antioxidant (net AUC). The Trolox antioxidant standard curve was applied to obtain the ORAC antioxidant index of the sample.

### Determination of ACE inhibitory activity

The modified approach of Cushman and Cheung^68^ was used to analyze ACE inhibitory activity. Briefly, the preincubation of 20 µL of sample and 30 µL of ACE solution (60 mU/mL) was performed at 37 °C for 5 min. After adding 50 µL of the substrate (7.6 mmol/L HHL in 50 mmol/L sodium borate buffer containing 300 mmol/L NaCl at pH 8.3), the 30-min incubation of the mixture was performed at ambient temperature. The reaction was ceased with 100 µL of 1.0 mol/L HCl. RP-HPLC was employed to determine the amount of HA liberated by ACE on an Inertsil ODS-SP C18 column (4.6 mm × 150 mm, Shimadzu, Kyoto, Japan). The peak area was subject to the calculation of ACE inhibitory activity.

### Isolation of WOP with RP-HPLC

An RP-HPLC system (Shmadzu, Kyoto, Japan) with an XBridge BEH130 C18 column (4.6 × 250 mm, Waters, USA) was used to separate WOP. Eluent A (Milli-Q water with 0.1% (v/v) TFA) and eluent B (80% (v/v) acetonitrile with 0.1% (v/v) TFA) were used for gradient elution with the following program: 0–15% B for 0–50 min, 15–40% B for 50–120 min, and 40–80% B for 120–140 min. The flow rate was 0.6 mL/min. The effluent was monitored at 220 nm. Fifty microliters of the 10 mg/mL sample was injected. The separation procedure was repeated successively for six times using automatic collector (Fraction Collecter10A, Shmadzu, Kyoto, Japan) by setting up the automatic collection time program. For further analysis, the fractions from the RP-HPLC system were freeze-dried.

### Recognition of active peptides

A quadrupole time-of-flight mass spectrometer (Q-TOF2; Micromass Co., Manchester, UK) with an electrospray ionization (ESI) source was employed to analyze the molecular masses and amino acid sequences of peptides. The freeze-dried RP-HPLC fractions were dissolved in distilled water, and subjected to the ESI-Q-TOF2 mass spectrometer. The collision energy was 0–200 V. The flow rate was 5 µL/min. The ionization potential was 3,000 V in positive mode. The atomization gas and collision gas were N_2_ and Ar, respectively. Firstly, the ESI–MS spectrogram was obtained by first-order mass spectrometry. The ions to be measured were selected from the ESI–MS spectrogram, and then analyzed to obtain the ESI–MS/MS spectrogram. The mass spectrum was analyzed using MassLynX software (Waters, USA).

### Peptide syntheses

Peptides were synthesized using the conventional solid-phase synthesis (fluorenyl methoxycarbonyl (Fmoc) strategy) with a peptide synthesizer (model 90, AAPPTec, USA) by GL Biochem (Shanghai) Ltd. The synthesized peptides were purified and detected by HPLC using a C18 column (ProStar, Varian Co., USA). The molecular masses of the peptides were determined by a mass spectrometer (Voyager DE-Pro, ABI, USA). The synthesized peptides with the purity of higher than 98% were stored at − 80 °C until use^2^.

### Quantitative analysis of active peptides

HPLC–MS/MS was employed to quantitatively analyze the peptides in WOP. A Dionex Ultimate 3000 HPLC system (Thermo Scientific, Waltham, MA, USA) was applied to separate peptides on an MSLAB HP-C18 column (150 mm × 4.6 mm) by a 10 min gradient elution at a flow rate of 1.0 mL/min. Eluent A (water with 2 mmol/L ammonium formate) and eluent B (acetonitrile with 2 mmol/L ammonium formate) were used to elute peptides. Ten microliters of the sample was injected. An API 3200 Q TRAP HPLC–MS/MS (Applied Biosystems, Foster City, CA, USA) with an ESI source was used to perform tandem mass spectrometry. The collision energy was 30 eV. The ionspray voltage was 4500 V in positive mode. The atomization gas and auxiliary gas were 45 and 50 psi, respectively. The atomization temperature was 550 ℃. The mass spectrum was analyzed using Analyst Software 1.5 (Applied Biosystems, USA). WOP and the synthesized peptides were dissolved in distilled water, and subjected to HPLC–MS/MS. The standard curve equation of each peptide was obtained. Then, the quantification of each peptide in WOP was performed and measured.

### Statistical analysis

All experiments were conducted in triplicate, and the data are expressed as means ± standard deviations.
